# Outscoring “Fire and Forget”? Current Practice of Lipid Management in Kidney Transplant Recipients

**DOI:** 10.3389/ti.2025.14600

**Published:** 2025-08-29

**Authors:** Eric Amelunxen, Roland Schmitt, Laura Katharina Sievers

**Affiliations:** Department of Nephrology and Hypertension, University Hospital Schleswig-Holstein Campus Kiel, Christian-Albrechts-University, Kiel, Germany

**Keywords:** LDL-C, hyperlipidaemia, mortality, kideny transplantation, cardiometabolic disease

Dear Editors,

Kidney transplant recipients (KTR) have a very high cardiovascular risk and cardiovascular disease (CVD) is the major cause of death contributing to decreased life expectancy in this population [1]. Dyslipidemia is a major modifiable cardiovascular risk factor, and effective management can significantly improve patient outcomes [2]. The current 2013 KDIGO guideline advocates for statin therapy and recommends a “fire and forget” strategy without specification of low-density lipoprotein cholesterol (LDL-C) target levels [3]. In contrast, the European Society of Cardiology (ESC) guideline recommends a “treat to target” approach with LDL-C treatment goals based on risk stratification [4]. So far, evidence-based recommendations for optimal lipid management in the vulnerable KTR population are missing. In order to get a clear view on current practice in KTR lipid management, we conducted a survey among all transplant centers within the Eurotransplant (ET) network. Of 107 centers in total (n = 81 DACH region and n = 26 international), 18 centers responded (response rate 17%, with 13 German and 5 international ET-Centers). 61% (n = 11) of the responders reported having a standardized protocol for lipid management. The treatment goals for LDL-C varied significantly. In total, 77% reported a defined target range. 44% referenced the ESC guideline high risk treatment goals (Figure 1). The most common LDL-C target ranges were <55 mg/dL (1.4 mmol/L) and 70–100 mg/dL (1.4–1.8 mmol/L). Only 17% (n = 3) of centers had no specific treatment goal, as according to the KDIGO guidelines. A single center reported using individualized treatment goals that did not follow any guidelines or recommendations.

**FIGURE 1:**
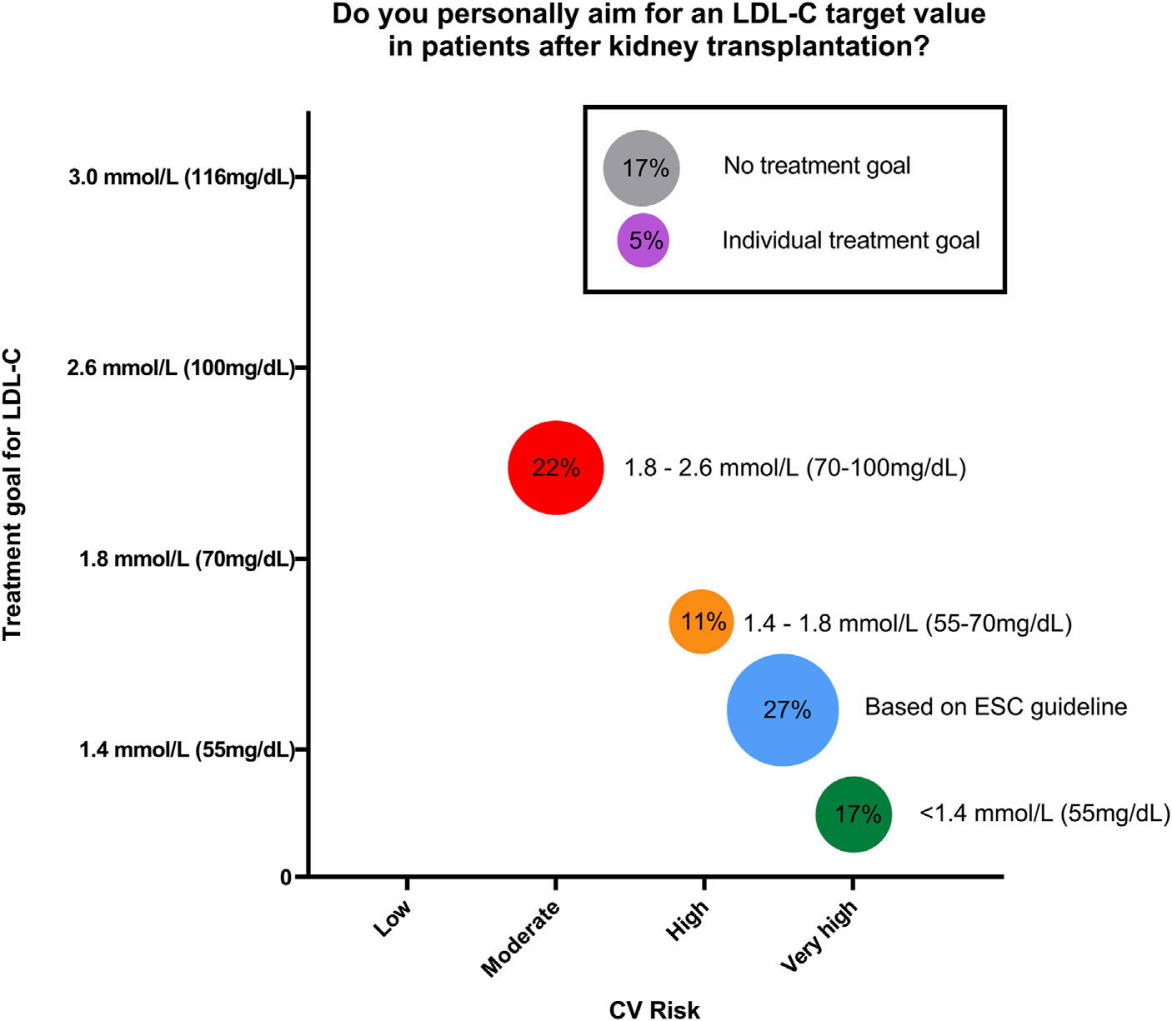
Schematic representation of answers from Eurotransplant Kidney transplant centers to the question “Do you personally aim for an LDL-C target value in patients after kidney transplantation” (n = 18; circle size correlates with percentage).

Therefore, our survey reveals striking heterogeneity in the management of dyslipidemia in KTRs. Based on the ambiguous recommendations of KDIGO and ESC and the lack of randomized controlled trials evaluating LDL-C target values in KTR, this is not surprising. Interestingly, our results suggest that many centers are moving towards targeted approach, congruent with the ESC guidelines. As a notable constraint, 39% (n = 7) of centers report to not have a standard protocol, indicating a substantial proportion of patients receive treatment dependent on physician’s personal preferences.

We argue that KTRs deserve an optimal standardized therapy of their CV risk factors: Most of these patients have a years-long history of CKD and dialysis, which are associated with accelerated atherosclerosis and CVD remains the leading cause of death in KTR.

In the ALERT Trial, Fluvastatin showed effective risk reduction for cardiac death and non-fatal myocardial infarction while adverse events to statin therapy were not reported [5]. The results suggest that beneficial results of statin therapy from non-transplant cohorts are projectable to the KTR population. Our data underlines that most practitioners share this view, albeit studies comparing a targeted vs. non-targeted approach are missing.

Importantly, targeted therapy may require higher statin doses or combination therapy with ezetimibe, bempedoic acid and/or PCSK9-inhibitors, raising the question for cost-benefit analysis in this setting.

Taken together, there is significant variability in lipid management practices among transplant centers regarding LDL-C target values. Our survey did not capture patient outcomes nor detailed information on the lipid-lowering therapies used.

The majority of transplant centers does not stick to the “fire-and-forget” strategy advocated by the KDIGO but KTR lipid management is oriented at the ESC guidelines. Apparently, real-world KTR lipid management has outpaced the current KDIGO recommendations, underscoring the need for further research and updated nephrological guidelines for lipid management in KTR. Standardized protocols could improve patient outcomes across centers as the management of CVD remains the essential challenge in this high-risk population.

## Data Availability

The original contributions presented in the study are included in the article/supplementary material, further inquiries can be directed to the corresponding author.
